# Poly(rC)-Binding Protein 2 Does Not Directly Participate in HCV Translation or Replication, but Rather Modulates Genome Packaging

**DOI:** 10.3390/v16081220

**Published:** 2024-07-30

**Authors:** Sophie E. Cousineau, Carolina Camargo, Selena M. Sagan

**Affiliations:** 1 Department of Microbiology & Immunology, McGill University, Montreal, QC H3A 2B4, Canada; 2 Department of Microbiology & Immunology, University of British Columbia, 2350 Health Science Mall, Room 4.520, Vancouver, BC V6T 1Z3, Canada

**Keywords:** hepatitis C virus (HCV), poly(rC)-binding protein 2 (PCBP2), hnRNP E2, translation, replication, assembly

## Abstract

The hepatitis C virus (HCV) co-opts many cellular factors—including proteins and microRNAs—to complete its life cycle. A cellular RNA-binding protein, poly(rC)-binding protein 2 (PCBP2), was previously shown to bind to the hepatitis C virus (HCV) genome; however, its precise role in the viral life cycle remained unclear. Herein, using the HCV cell culture (HCVcc) system and assays that isolate each step of the viral life cycle, we found that PCBP2 does not have a direct role in viral entry, translation, genome stability, or HCV RNA replication. Rather, our data suggest that PCBP2 depletion only impacts viral RNAs that can undergo genome packaging. Taken together, our data suggest that endogenous PCBP2 modulates the early steps of genome packaging, and therefore only has an indirect effect on viral translation and RNA replication, likely by increasing the translating/replicating pool of viral RNAs to the detriment of virion assembly.

## 1. Introduction

Hepatitis C virus (HCV) is an enveloped, positive-sense RNA virus of the *Flaviviridae* family that typically causes persistent liver infection [1]. Its ~9.6 kb genome contains a single open reading frame flanked by highly structured 5′ and 3′ untranslated regions (UTRs). The 5′ UTR contains stem-loop (SL) structures necessary for RNA replication (SLI-SLII) and translation (SLII-IV), while the 3′ UTR (composed of a hypervariable region, a poly(U/UC) tract, and a highly conserved 3′ X-tail) is implicated in viral replication. In the 5′ UTR, SLII-IV form an internal ribosomal entry site (IRES) that drives translation of the viral polyprotein, which is processed into ten mature viral proteins, including three structural proteins (core, E1 and E2 glycoproteins), and seven non-structural proteins (p7, NS2, NS3, NS4A, NS4B, NS5A and NS5B) [2,3]. The structural proteins form the nucleocapsid and viral envelope, while the non-structural proteins participate in polyprotein processing, genome replication, immune evasion, and virion assembly [4,5,6,7,8,9].

The poly(rC)-binding protein 2 (PCBP2) is one of the three most abundant cellular RNA-binding proteins with a strong affinity for poly(rC), along with its paralogs hnRNP K and PCBP1 [10,11]. PCBP2 is a multifunctional protein that can bind hundreds of cellular RNAs, including its own mRNA transcript [12]. Depending on the context of its binding site and binding partners, PCBP2 can modulate the stability and/or translation of its mRNA targets [13,14]. Beyond its cellular mRNA interactions, PCBP2 has been reported to interact with several viral RNAs, and is implicated in regulation of viral translation, RNA stability, replication and/or viral assembly [15,16,17,18,19,20,21,22,23]. PCBP2’s role in the poliovirus (PV) life cycle is particularly well-characterized, where it mediates the switch from translation to replication [24,25,26,27,28,29]. Specifically, PCBP2 is an essential IRES *trans*-acting factor, part of the complex that circularizes the PV genome to initiate viral RNA replication, and viral protease-mediated PCBP2 cleavage triggers the switch from translation to replication.

In previous siRNA screens, PCBP2 knockdown was found to initially enhance HCV titers and viral RNA levels, followed by a sharp decline in viral RNA accumulation and infectious particle production [30]. However, the precise mechanisms by which PCBP2 modulates HCV RNA accumulation and viral particle production have remained elusive, as studies examining viral translation and RNA replication using different experimental systems arrived at contradictory conclusions [31,32,33,34,35,36,37,38,39]. Identification of PCBP2 binding sites in the HCV genome were mapped by cross-linking immunoprecipitation (iCLIP), which identified six conserved PCBP2 binding sites across two HCV genotypes [40]. In agreement with previous studies that identified binding sites in the HCV 5′ and 3′ UTRs, two binding sites were identified in the 5′ UTR (near SLI, and over the initiation codon in SLIV of the IRES), three sites were identified in the polyprotein-coding region (in the *core*, *E2* and *NS5B* genes), and one site is located in the 3′ UTR (overlapping the variable region and poly(U/UC)-tract) [33,40,41,42]. However, despite the identification of these PCBP2 interaction sites, the role of PCBP2 in the HCV life cycle remains unclear. Thus, herein we aimed to clarify the role of PCBP2 in the HCV life cycle.

Using cell culture-derived HCV (HCVcc), we found that endogenous PCBP2 was important for optimal HCV RNA accumulation in cell culture. By examining individual steps of the viral life cycle, we ruled out a direct role for PCBP2 in viral entry, translation, genome stability, and viral RNA replication. Rather, we discovered that only viral RNAs that undergo the early steps of viral genome packaging were sensitive to PCBP2 knockdown, suggesting that endogenous PCBP2 normally affects HCV assembly, and might only indirectly affect viral translation and viral RNA accumulation by modulating the viral RNAs available to engage in each of these processes.

## 2. Materials and Methods

### 2.1. Cell Culture

Huh-7.5 human hepatoma cells were provided by Dr. Charlie Rice (Rockefeller University) and maintained in complete media: Dulbecco’s Modified Eagle Media (DMEM) supplemented with heat-inactivated 10% fetal bovine serum (FBS), 2 mM L-glutamine, and 1X MEM non-essential amino acids. Human embryonic kidney cells (293T) were provided by Dr. Martin J. Richer (McGill University) and HeLa cervical epithelial adenocarcinoma cells were obtained from the ATCC (CCL-2) and were both maintained in DMEM supplemented with 10% FBS. All cells were maintained at 37 °C/5% CO_2_ and were routinely screened for mycoplasma contamination.

### 2.2. Plasmids and Viral RNAs

Plasmids encoding a firefly luciferase (FLuc) reporter gene under the translational control of a poliovirus IRES (PV IRES, nt 71–732), HCV IRES (nt 40–372) or encephalomyocarditis virus IRES (EMCV IRES, nt 281–848) were a kind gift from Drs. Yuri Svitkin and Nahum Sonenberg [43]. These plasmid templates were linearized with *BamHI* and in vitro transcribed using T7 RNA polymerase as previously described [44]. The Renilla luciferase (RLuc) mRNA was transcribed from the pRL-TK plasmid (Promega, Madison, WI, USA) linearized using *BglII* and in vitro transcribed using the mMessage mMachine T7 Kit (Life Technologies, Carlsbad, CA, USA) according to the manufacturer’s instructions.

The pJFH-1_T_ plasmid encodes a cell culture-adapted Japanese Fulminant Hepatitis (JFH-1; HCV genotype 2a) with three adaptive mutations that increase viral titers in cell culture [45]. The bicistronic pSGR FLuc WT plasmid bears a subgenomic replicon derived from JFH-1, where the core through NS2 region has been replaced by a firefly luciferase (FLuc) reporter gene and with an EMCV IRES, which drives the translation of the NS3 through NS5B proteins [46] and was provided by Dr. Ralf Bartenschlager. The pJ6/JFH1 FL RLuc WT (“RLuc-wt”) and pJ6/JFH-1 FL RLuc GNN (“RLuc-GNN”) plasmids bear full-length viral sequences derived from the J6 (structural genes) and JFH-1 (nonstructural genes) isolates of HCV, with a *Renilla* luciferase (RLuc) reporter inserted between the p7 and NS2-coding regions. RLuc-GNN also bears an inactivating GNN mutation within the NS5B RNA polymerase active site [5]. The pJ6/JFH-1 mono RLuc-NS2 (“Δcore-p7”) and pJ6/JFH-1 E1-p7 del (“ΔE1-p7”) plasmids—truncated versions of the *Renilla* reporter virus with deletions of structural genes through p7—were provided by Dr. Joyce Wilson (University of Saskatchewan, Saskatoon, SK, Canada) [47]. The pJ6/JFH Δcore (“ΔCore”) plasmid consists of a truncated version of the *Renilla* reporter virus with a deletion of the core-coding gene that retained the first 15 codons (necessary for a functional HCV IRES) and the final 14 codons (which orient the E1 protein in the ER) of the core-coding sequence. To clone ΔCore, the *EcoRI* to *KpnI* fragment was subcloned to a temporary plasmid and PCR-amplified with Q5 high fidelity DNA polymerase (NEB) using the dCore-BamHI-FW (5′-TTT CTG GAT CCT TGC TGG CCC TGC TGT CCT GCA TC-3′) and dCore-BamHI-RV (5′-GGG CGG GAT CCG GTG TTT CTT TTG GTT TTT CTT TGA G-3′) primers, and was auto-ligated after *BamHI* digestion. The *EcoRI* to *KpnI* fragment was then subcloned back into the parental pJ6/JFH-RLuc plasmid. The pJ6/JFH-1 FL RLuc-NS5A-GFP (“NS5A-GFP”) plasmid, a full-length *Renilla* reporter virus with a GFP insertion within the NS5A domain III, was subcloned as previously described [48].

To make uncapped viral RNAs, all plasmid templates were linearized with *XbaI* and were in vitro transcribed with T7 RNA polymerase (NEB). Briefly, 1 µg of linear template DNA was incubated at 30 °C for 1 h with 200 U T7 RNA polymerase, 1 mM each of ATP, CTP, and UTP, 1.2 mM GTP, and 50 U RiboLock RNAse inhibitor (ThermoFisher Scientific, Waltham, MA, USA), followed by a 15 min DNaseI (NEB) digestion at 37 °C. The firefly luciferase (FLuc) mRNA was transcribed from the Luciferase T7 Control DNA plasmid (Promega) linearized using *XmnI* and in vitro transcribed using the mMessage mMachine T7 Kit (Life Technologies) according to the manufacturer’s instructions.

### 2.3. Generation of Infectious HCV Stocks

To generate viral stocks, 30 µg of in vitro transcribed JFH-1_T_ RNA was transfected into Huh-7.5 cells using the DMRIE-C reagent (Life Technologies) according to the manufacturer’s instructions. Four days post-transfection, infectious cell supernatants were passed through a 0.45 µm filter and infectious viral titers were determined by focus-forming unit assay [45]. Infectious virus was amplified for two passages through Huh-7.5 cells at a multiplicity of infection (MOI) of 0.05. Viral stocks were aliquoted and stored at −80 °C until use.

### 2.4. Focus-Forming Unit (FFU) Assays

One day prior to infection, 8-well chamber slides (ThermoFisher Scientific, Waltham, MA, USA) were seeded with 4 × 10^5^ Huh-7.5 cells/well. Infections were performed with 10-fold serial dilutions of viral samples in 100 µL for 4 h, after which the supernatant was replaced with fresh media. Three days post-infection, slides were fixed in 100% acetone and stained with anti-HCV core antibody (1:100, clone B2, Anogen, Mississauga, ON, Canada), and subsequently with the AlexaFluor-488-conjugated anti-mouse antibody (1:200, ThermoFisher Scientific, Waltham, MA, USA) for immunofluorescence analysis. Viral titers are expressed as the number of focus-forming units (FFU) per mL. Extracellular virus titers were determined directly from cell supernatants.

### 2.5. MicroRNAs and siRNA Sequences

siGL3 (siCTRL): 5′-CUU ACG CUG AGU ACU UCG AUU-3′, siGL3*: 5′-UCG AAG UAC UCA GCG UAA GUU-3′, miR122_p2-8_ (siCTRL for luciferase experiments): 5′-UAA UCA CAG ACA AUG GUG UUU GU-3′, miR122_p2-8_*: 5′-AAA CGC CAU UAU CUG UGA GGA UA-3′ [49], siPCBP2-1: 5′-UCC CUU UCU GCU GUU CAC CUU-3′, siPCBP2-1*: 5′-GGU GAA CAG CAG AAA GGG AUU-3′, siPCBP2-2: 5′-GGA CAG UAU GCC AUU CCA CUU-3′, and siPCBP2-2*: 5′-GUG GAA UGG CAU ACU GUC CUU-3′ [30] were all synthesized by Integrated DNA Technologies.

All microRNA and siRNA duplexes were diluted to a final concentration of 20 µM in RNA annealing buffer (150 mM HEPES pH 7.4, 500 mM potassium acetate, 10 mM magnesium acetate), annealed at 37 °C for 1 h and stored at −20 °C. Annealed siPCBP2-1 and siPCBP2-2 were mixed together at a 1:1 ratio prior to transfection. For all knockdown experiments, 50 nM siRNA transfections were conducted 2 days prior to infection or electroporation of viral RNAs. Transfections were conducted using the Lipofectamine RNAiMAX (Invitrogen, Waltham, MA, USA) according to the manufacturer’s instructions with the modification that 20 µL of reagent were used to transfect a 10-cm dish of cells.

### 2.6. HCV and VSV Pseudoparticles (HCVpp and VSVpp)

HCVpp consisting of a Firefly luciferase reporter lentiviral vector pseudotyped with the HCV E1 and E2 glycoprotein (from the H77, a genotype 1a strain) were a kind gift from Dr. John Law (University of Alberta) [50]. To generate lentiviral vectors pseudotyped with the VSV-G glycoprotein (VSVpp), a 90% confluent 10-cm dish of 293T cells were transfected with 10 µg pPRIME-FLuc, 5 µg psPAX.2, and 2.5 µg pVSV-G plasmid with 10 µL Lipofectamine 2000 (Invitrogen) diluted in 4 mL Opti-MEM. The media was changed 4, 20, and 28 h post-transfection. At 48 h post-transfection, the cell culture media was passed through a 0.45 µm filter and stored at −80 °C.

To assay for cell entry, HCVpp and VSVpp were diluted 1/3 in dilution media (1X DMEM, 3% FBS, 100 IU penicillin and 100 µg/mL streptomycin) with 20 mM HEPES and 4 µg/µL polybrene, and then introduced to Huh-7.5 cells by spinoculation at 1200 rpm for 1 h at room temperature. The cells were left to recover at 37 °C for at least 5 h before the pseudoparticle-containing media was changed for fresh complete Huh-7.5 media. In parallel, cells seeded in a 6-well plate were transfected with 1 µg of pPRIME-FLuc plasmid using Lipofectamine 2000 (Invitrogen) according to the manufacturer’s instructions. Three days post-spinoculation and transfection, cells were lysed in passive lysis buffer (Promega) and firefly luciferase activity was assayed using the Dual Reporter Luciferase kit (Promega).

### 2.7. Infections

Three days prior to infection, 10-cm dishes were seeded with 5 × 10^5^ Huh-7.5 cells, which were transfected with siRNA duplexes on the following day. On the day of infection, each 10-cm dish—containing approximately 1 × 10^6^ cells—was infected with 5 × 10^4^ FFU of JFH-1_T_ diluted in 3 mL complete media. Four to five hours post-infection, each infected plate was split into three 10-cm dishes. Protein, RNA, and virus samples were collected three days post-infection.

### 2.8. Electroporations

For each electroporation, 6 × 10^6^ cells resuspended in 400–600 µL cold PBS were mixed with 5 µg of replication-competent viral RNA, or with 10 µg of nonreplicative GNN J6/JFH-1 RLuc RNA with 2 µg of FLuc mRNA, and electroporated in 4-mm cuvettes at 270 V, 950 µF, infinite resistance optimized for the Bio-Rad GenePulser XCell (Bio-Rad, Hercules, CA, USA). Electroporated cells were resuspended in complete Huh-7.5 media and transferred to 6-well plates for luciferase assays and protein expression analyses, or to a 10-cm dish to assess infectious virus production.

### 2.9. Western Blot Analysis

To collect total intracellular protein samples, cells were lysed in RIPA buffer (150 mM sodium chloride, 1% NP-40, 0.5% sodium deoxycholate, 0.1% SDS, 50 mM Tris pH 8.0) supplemented with Complete Protease Inhibitor Cocktail (Roche, Basel, Switzerland) and frozen at −80 °C. Cellular debris was pelleted by centrifugation at 16,000× *g* for 30 min at 4 °C, and the supernatant protein concentration was quantified by BCA Protein Assay (ThermoScientific). Ten micrograms of protein was separated on 10–12% SDS-PAGE gels prior to transfer onto Immobilon-P PVDF membranes (Millipore, Burlington, MA, USA). Membranes were blocked in 5% milk and incubated overnight with primary antibodies diluted in 5% BSA: mouse anti-PCBP2 (clone 5F12, H00005094-M07 Abnova, Taipei City, Tapei, diluted 1:20,000); rabbit anti-actin (A2066, Sigma Aldrich, St. Louis, MI, USA, 1:20,000); mouse anti-HCV core (clone B2, MO-I40015B Anogen, Mississauga, ON, Canada, 1:7500); mouse anti-JFH-1 NS5A (clone 7B5, BioFront Technologies, Tallahassee, FL, USA, 1:10,000). Blots were incubated for 1 h with HRP-conjugated secondary antibodies diluted in 5% milk: anti-mouse (HAF007, R&D Systems, Minneapolis, MN, USA, 1:25,000); anti-rabbit (111-035-144, Jackson ImmunoResearch Laboratories, West Grove, PA, USA, 1:50,000) and visualized using enhanced chemiluminescence (ECL Prime Western Blotting Detection Reagent, Fisher Scientific, Waltham, MA, USA).

### 2.10. RNA Isolation and Northern Blot Analysis

Total RNA was harvested using TRIzol Reagent (ThermoFisher Scientific) according to the manufacturer’s instructions. Ten micrograms of total RNA were separated on a 1% agarose gel containing 1X 3-(N-morpholino)propanesulfonic acid (MOPS) buffer and 2.2 M formaldehyde and transferred to a Zeta-probe membrane (Bio-Rad) by capillary transfer in 20X SSC buffer (3 M NaCl, 0.3 M sodium citrate). Membranes were hybridized in ExpressHyb Hybridization Buffer (ClonTech, Mountain View, CA, USA) to random-primed ^32^P-labeled DNA probes (RadPrime DNA labelling system, Life Technologies, Carlsbad, CA, USA) complementary to HCV (nt 84–374) and γ-actin (nt 685–1171). Autoradiograph band densities were quantified using Fiji (ImageJ2 2.3.0) [51].

### 2.11. RT-qPCR Analysis

The iTaq Universal Probes One-Step kit (Bio-Rad) was used to perform duplex assays probing for the HCV genome (NS5B-FW primer: 5′-AGA CAC TCC CCT ATC AAT TCA TGG C-3′; NS5B-RV primer: 5′-GCG TCA AGC CCG TGT AAC C-3′; NS5B-FAM probe: 5′-ATG GGT TCG CAT GGT CCT AAT GAC ACA C-3′) [48] and the GAPDH loading control (PrimePCR Probe assay with HEX probe, Bio-Rad). Each 20 µL reaction contained 500 ng of total RNA, 1.5 µL of the HCV primers and probe, and 0.5 µL of the GAPDH primers and probe. RT-PCR reactions were conducted in a CFX96 Touch Deep Well Real-Time PCR system (Bio-Rad). Genome copies were calculated against a genomic RNA standard curve, and fold-differences in gene expression were calculated using the 2^−∆∆Ct^ method [52].

### 2.12. Luciferase Assays

For IRES-mediated translation assays, HeLa cells were plated at a density of 5 × 10^5^ cells/10-cm dish and transfected with 50 nM of siPCBP2 or siCTRL siRNA duplexes, as described in the main text’s methods. The next day, transfected cells were seeded into 6-well plates at a density of 2.5 × 10^5^ cells per well. On the second day post-siRNA transfection, each well was co-transfected with 1.5 µg of IRES-FLuc IVT and 2.5 µg of RLuc mRNA, resuspended into 1 mL OptiMEM with 2.5 µL of Lipofectamine 3000 (Invitrogen). Four hours post-transfection, cell culture media was changed for DMEM with 10% FBS. The next day, each well was harvested in 100 µL of 1X passive lysis buffer (Promega). For translation and replication assays, cells were washed in PBS and harvested in 100 µL of 1X passive lysis buffer (Promega). The Dual-Luciferase Assay Reporter Kit (Promega) was used to measure both *Renilla* and firefly luciferase activity according to the manufacturer’s instructions with the modification that 25 µL of reagent were used with 10 µL of sample. All samples were measured in triplicate.

### 2.13. Data Analysis

Statistical analyses were performed using GraphPad Prism v9 (GraphPad Software, La Jolla, CA, USA). Statistical significance was determined by paired *t*-test to compare results obtained from multiple experiments, and by two-way ANOVA with Geisser-Greenhouse and Bonferroni corrections when more than two comparisons were applied at once.

## 3. Results

### 3.1. PCBP2 Is Required for Optimal HCVcc Accumulation in Cell Culture

**Figure 1:**
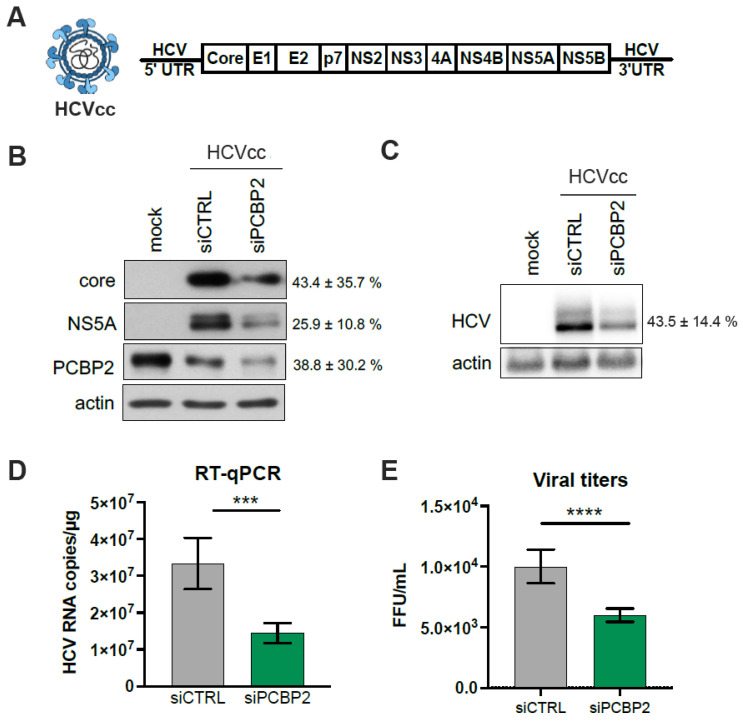
PCBP2 is required for optimal HCV RNA accumulation and infectious particle production in cell culture. (**A**) Schematic representation of the HCVcc (JFH-1_T_) infectious particles and genomic RNA used in infections. (**B**–**E**) Huh-7.5 cells were transfected with siPCBP2 or with siCTRL 2 days prior to infection with JFH-1_T_ (MOI = 0.05). Whole cell lysates, total RNA, and intracellular and extracellular infectious virions were harvested at 3 days post-infection. (**B**) Viral protein expression analysis by Western blot. (**C**) Viral RNA accumulation analysis by Northern blot, (**D**) quantification by RT-qPCR, and (**E**) extracellular (secreted) virus titers, quantified by FFU assay. All data are representative of three independent biological replicates, and error bars represent the standard deviation of the mean. *p*-values were calculated by paired *t*-test (*** *p* < 0.001; **** *p* < 0.0001).

A previous siRNA screen identified PCBP2 as an important cellular factor for optimal HCV replication in cell culture [30]. To further investigate the role of PCBP2, we knocked down endogenous PCBP2 in Huh-7.5 cells, infected with the JFH-1_T_ strain (HCVcc) at an MOI = 0.05, and assessed viral protein expression, viral RNA accumulation, and infectious virion production (Figure 1). Notably, transfecting cells with a mix of two anti-PCBP2 siRNAs generally led to a transient increase in PCBP2 protein levels at day 1 post-transfection, followed by a sustained knockdown from days 2–5 (Figure S1). As such, all HCV infections were carried out at day 2 post-siRNA transfection. Endogenous PCBP2 knockdown resulted in an approximately 2.3-fold decrease in viral protein, RNA accumulation, and viral titers in Huh-7.5 cells (Figure 1). Thus, in line with previous studies, endogenous PCBP2 is necessary for optimal HCV RNA accumulation in cell culture, although the precise step(s) of the viral life cycle modulated by PCBP2 remain unclear.

### 3.2. PCBP2 Knockdown Has No Effect on HCV Entry

**Figure 2:**
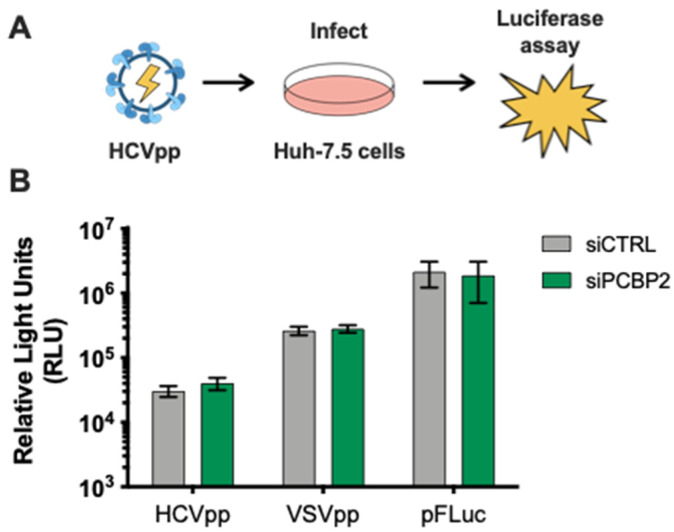
PCBP2 knockdown has no effect on HCV entry. (**A**) Schematic representation of HCV luciferase pseudoparticle (HCVpp) assay. (**B**) Two days post-siRNA transfection, cells were spinoculated with luciferase reporter pseudoparticles bearing the HCV E1/E2 glycoproteins (HCVpp) or the VSV-G glycoprotein (VSVpp). In parallel, cells were transfected with a firefly luciferase expression plasmid. Samples were harvested 3 days post-infection/transfection and analyzed by luciferase assay. The HCVpp and pFLuc data are representative of three independent replicates, while the VSVpp data are representative of two independent replicates. Error bars represent the standard deviation of the mean.

To clarify PCBP2’s role in the HCV life cycle, we used assays to specifically examine each step of the viral life cycle. First, we explored if PCBP2 knockdown had any effect on viral entry using the HCV pseudoparticle (HCVpp) system, which consists of lentiviral vectors with a firefly luciferase reporter gene pseudotyped with the HCV E1 and E2 glycoproteins (Figure 2) [50]. HCVpp enters cells by clathrin-mediated endocytosis after engaging with HCV-specific entry receptors; as such, we used vesicular stomatitis virus (VSV) pseudoparticles (VSVpp) as a control for effects on clathrin-mediated endocytosis. Additionally, to verify that PCBP2 knockdown did not affect luciferase reporter gene expression, we assessed firefly luciferase expression from cells directly transfected with a FLuc reporter plasmid. In all cases, we found that PCBP2 knockdown had no impact on luciferase activity (Figure 2). This suggests that endogenous PCBP2 does not affect FLuc reporter gene expression, clathrin-mediated endocytosis, or HCVpp entry.

### 3.3. PCBP2 Knockdown Has No Effect on HCV IRES-Mediated Translation or Genome Stability

**Figure 3:**
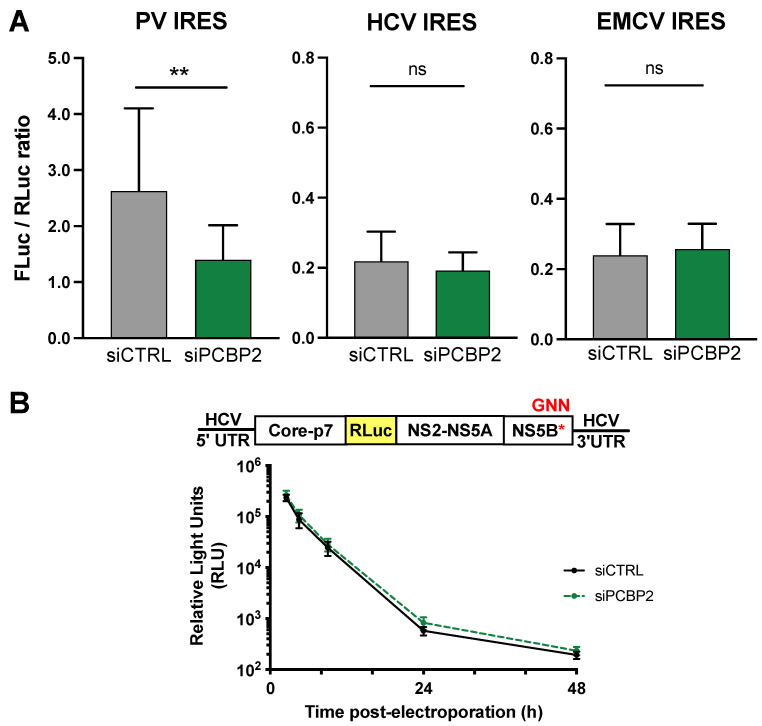
PCBP2 knockdown has no effect on HCV IRES-mediated translation or genome stability. (**A**) Two days post-siRNA transfection, HeLa cells were co-transfected with 2.5 µg of capped *Renilla* luciferase mRNA and 1.5 µg of RNAs comprised of a firefly luciferase (FLuc) gene under the control of either the PCBP2-sensitive poliovirus (PV) IRES, the PCBP2-insensitive encephalomyocarditis virus (EMCV) IRES, or the hepatitis C virus (HCV) IRES. Total protein samples were harvested in passive lysis buffer one day later, and luciferase activity was determined using the Dual Luciferase Reporter Assay kit. The IRES-mediated translation signal (FLuc) was normalized to the transfection efficiency control (RLuc) signal. Bars represent the mean ± standard deviation of four independent replicates. *p*-values were determined by paired *t*-test (** *p* < 0.01). (**B**) Two days post-siRNA transfection, Huh-7.5 cells were co-electroporated with J6/JFH-RLuc-GNN reporter RNA and an FLuc control mRNA, and luciferase activity was monitored at 2, 4, 6, 24 and 48 h timepoints post-electroporation. Data are representative of three independent biological replicates. Error bars represent the standard deviation of the mean. No statistically significant differences were found by paired *t*-test or two-way ANOVA.

PCBP2 is a known IRES *trans*-acting factor for several cellular and viral IRESes [17,53,54,55]. However, it is still unclear whether PCBP2 affects HCV translation, as studies using a variety of experimental systems have arrived at contradictory conclusions [31,32,33,34,35,36,37,38,39]. Thus, to directly test PCBP2’s effect on viral translation, we assessed HCV IRES-mediated translation in isolation as well as in the context of a full-length J6/JFH-1 RLuc reporter RNA with an inactivating mutation in the NS5B polymerase active site (Figure 3).

To assess HCV IRES-mediated translation in isolation, we performed PCBP2 knockdown in HeLa cells, and then introduced in vitro transcribed PV, encephalomyocarditis (EMCV) and HCV IRES-Firefly luciferase (FLuc) reporter RNAs as well as a capped *Renilla* luciferase (RLuc) control mRNA (Figure 3A). As previously reported, we observed a reduction in luciferase activity upon PCBP2 knockdown with the PV IRES, suggesting a reduction in PV IRES-mediated translation. However, we did not observe any significant effect of PCBP2 knockdown on either HCV or EMCV IRES-mediated translation (Figure 3A). This suggests that PCBP2 has no effect on HCV IRES-mediated translation in isolation.

Importantly, we and others have previously shown that miR-122 binds to the 5′ UTR of the HCV genome and promotes viral RNA stability and translation in human liver cells [44,47,49,56,57,58,59,60,61,62,63,64,65,66,67,68,69]. Interestingly, recent studies suggested that PCBP2 might compete with miR-122 for binding to a specific site in the HCV 5′ UTR, and in turn, inhibit translation to alter the balance between viral translation and replication [34,39,70]. As such, we also addressed the effect of PCBP2 knockdown on HCV IRES-mediated translation in the context of the HCV genome (including the complete 5′ UTR) in Huh-7.5 cells, where miR-122 is highly abundant (Figure 3B). To this end, we used a full-length J6/JFH-1 RLuc GNN reporter RNA with inactivating mutations (GDD → GNN) in the NS5B polymerase active site (Figure 3B). In this system, RLuc activity serves as a direct measure of HCV IRES-mediated translation, and over time this signal also serves as a proxy for viral RNA stability.

Intriguingly, we found that the siPCBP2 and siCTRL conditions had similar RLuc activity at all time points post-electroporation in Huh-7.5 cells (Figure 3B). This suggests that even in Huh-7.5 cells, where miR-122 is highly abundant, and in the context of a full-length viral genome, PCBP2 knockdown has no effect on HCV IRES-mediated translation. Furthermore, since we observed similar decay curves for both the siCTRL and siPCBP2 conditions, our data suggest that PCBP2 knockdown also does not significantly alter viral RNA stability. Thus, these results suggest that PCBP2 has no effect on either HCV IRES-mediated translation or viral genome stability.

### 3.4. PCBP2 Knockdown Has No Effect on Viral RNA Replication

**Figure 4:**
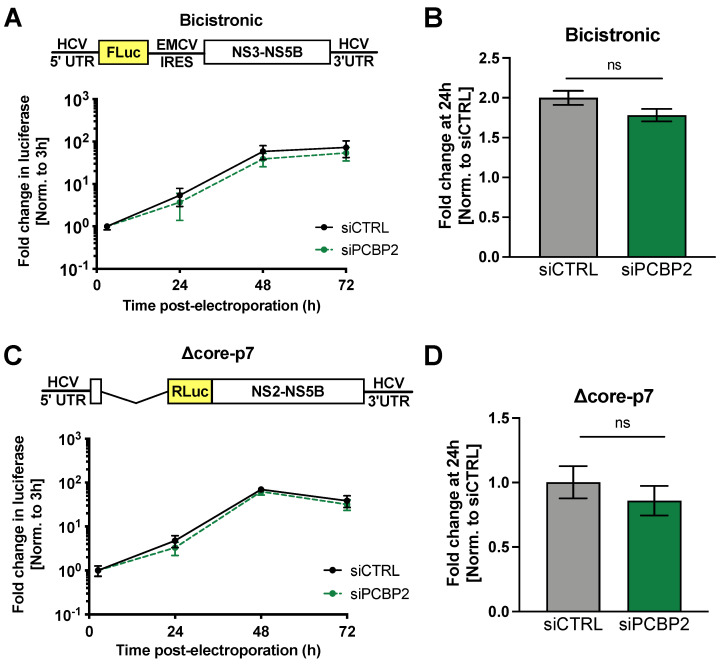
PCBP2 knockdown has no effect on HCV RNA replication. Two days post-siRNA transfection, Huh-7.5 cells were electroporated with 10 µg of (**A**,**B**) FLuc reporter bicistronic subgenomic replicon (bicistronic) as well as a capped RLuc mRNA (control), or (**C**,**D**) an RLuc reporter monocistronic subgenomic replicon (Δcore-p7) as well as a capped FLuc mRNA (control). Luciferase activity was monitored for three days post-electroporation. FLuc and RLuc values were normalized to the early timepoint (3 h), to control for disparities in electroporation efficiency between experiments. (**B**,**D**) Fold change in bicistronic (FLuc) or Δcore-p7 (RLuc) activity at the 24 h time point normalized to the siCTRL condition. Data are representative of three independent replicates; error bars represent the standard deviation of the mean. No statistically significant differences were found by two-way ANOVA.

PCBP2 has been previously reported to bind to and promote the replication of viral RNAs through interactions with their 5′ UTRs [19,55]. As such, we next examined if PCBP2 knockdown was able to modulate HCV RNA replication. To explore viral RNA replication in isolation (i.e., in the absence of viral packaging), we decided to explore viral RNA replication using a subgenomic replicon (Figure 4). First, we explored the effect of PCBP2 knockdown on a subgenomic bicistronic replicon, which lacks the *core* through *NS2*-coding region and whose expression of the NS3 through NS5B proteins, which carry out viral RNA replication, is driven by an EMCV IRES (Figure 4A,B). Surprisingly, given there have been several reports that PCBP2 modulates HCV RNA replication [34,38], we observed that the bicistronic replicon was completely insensitive to PCBP2 knockdown (Figure 4B).

While we had previously confirmed that the EMCV IRES is insensitive to PCBP2 knockdown (Figure 3A), to validate that this was not an artifact of the bicistronic nature of the subgenomic replicon used, we repeated this experiment using a monocistronic subgenomic replicon (Δcore-p7), lacking the *core* through *p7*-coding region (Figure 4C,D). Similarly, we observed that PCBP2 knockdown had no effect on the accumulation of this monocistronic subgenomic replicon RNA (Figure 4C,D). As such, PCBP2 knockdown has no effect on HCV RNA replication.

### 3.5. PCBP2 Sensitivity Maps to the HCV Core-Coding Region

As the subgenomic replicon data clearly indicate that PCBP2 knockdown has no impact on viral RNA replication, we reasoned that the PCBP2 effects we and others have observed (Figure 1) [34,38] may be related to either specific interactions between PCBP2 and the core-p7 region of the HCV RNA, or through a role in modulation of infectious particle production.

**Figure 5:**
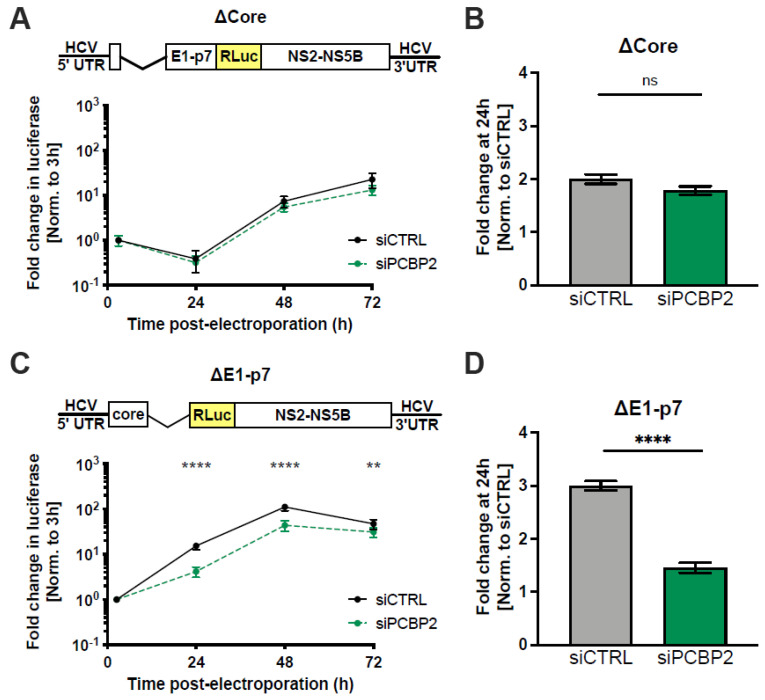
PCBP2 sensitivity maps to the *core* coding region. Two days post-siRNA transfection, Huh-7.5 cells were electroporated with (**A**,**B**) ΔCore J6/JFH-1 RLuc RNA and (**C**,**D**) ΔE1-p7 J6/JFH-1 RLuc RNA. (**A**,**C**) RLuc values were normalized to the early time point (3 h), to control for disparities in electroporation efficiency between experiments. (**B**,**D**) Fold change in ΔCore or ΔE1-p7 RLuc activity at the 24 h time point normalized to the siCTRL condition. Data are representative of three independent biological replicates; error bars represent the standard deviation of the mean. *p*-values were calculated by two-way ANOVA (** *p* < 0.01; **** *p* < 0.0001, data points without asterisks are not significant).

As a previous iCLIP study had suggested a high-confidence PCBP2 binding site in the *core* region of the HCV genome, we further mapped the viral gene requirements for PCBP2 sensitivity using subgenomic viral RNAs with deletions in the core- (ΔCore) or E1 through p7 (ΔE1-p7)-coding regions, specifically (Figure 5). Interestingly, while the ΔCore exhibited delayed replication kinetics, PCBP2 knockdown did not result in further impairment of viral RNA replication (Figure 5A,B). However, in line with what we observed using infectious virus (Figure 1), the ΔE1-p7 viral RNA was sensitive to PCBP2 knockdown, with an approximately 2.5-fold decrease in luciferase activity and concomitant reduction in viral RNA accumulation (Figure 5C,D). Moreover, this was clearly apparent at the RNA level when we compared viral RNA accumulation between the previous Δcore-p7 and ΔE1-p7 subgenomic replicons by both northern blot and RT-qPCR analyses (Figure S2). Taken together, these results indicate that only viral RNAs that contain the *core* gene are sensitive to PCBP2 knockdown; however, it remained unclear if the *core*-coding sequence (i.e., the RNA sequence) or the activity of the core protein was the determinant of PCBP2 sensitivity.

### 3.6. PCBP2 Modulates Viral Genome Packaging

**Figure 6:**
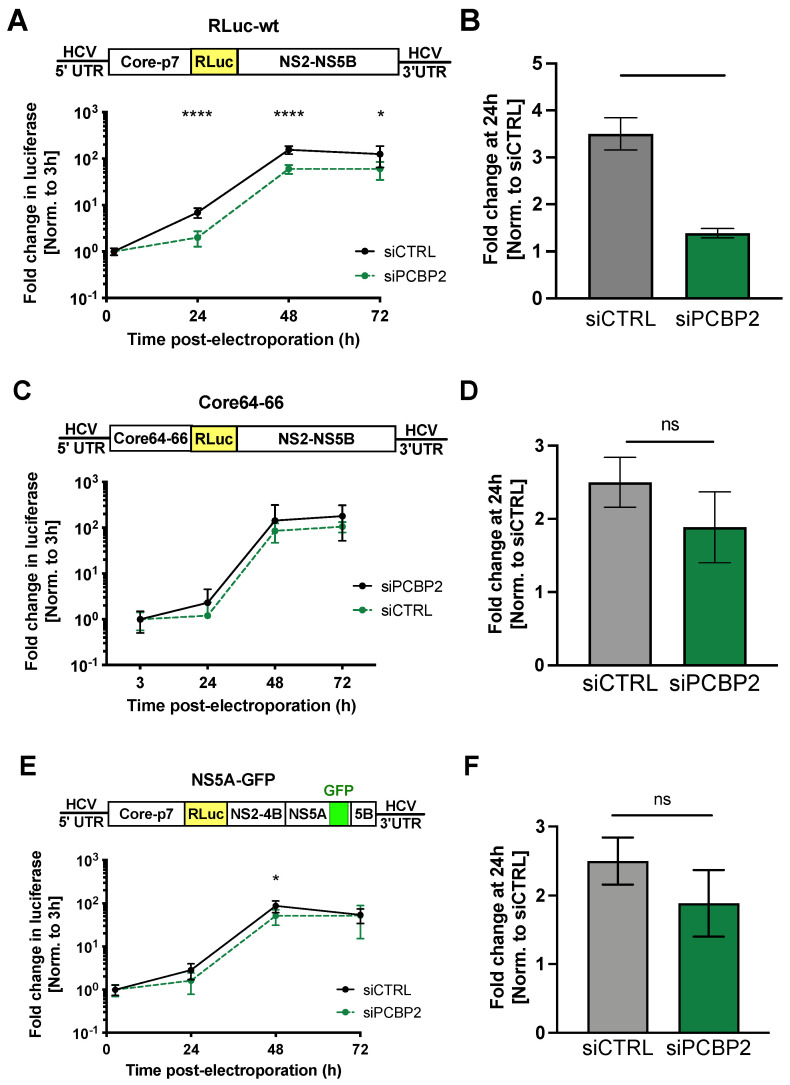
PCBP2 knockdown modulates viral genome packaging. Two days post-siRNA transfection, Huh-7.5 cells were electroporated with (**A**,**B**) full-length RLuc wild-type (RLuc-wt) J6/JFH-1 RNA, (**C**,**D**) full-length RLuc Core64–66 J6/JFH-1 RNA, and (**E**,**F**) full-length RLuc NS5A-GFP J6/JFH-1 RNA. RLuc values were normalized to the early time point (3 h), to control for disparities in electroporation efficiency between experiments. (**B**,**D**,**F**) Fold change in RLuc-WT, Core64–66 and NS5A-GFP at the 24 h time point normalized to the siCTRL condition. Data are representative of three independent biological replicates; error bars represent the standard deviation of the mean. *p*-values were calculated by two-way ANOVA (* *p* < 0.05; **** *p* < 0.0001, data points without asterisks are not significant).

We reasoned that PCBP2 might regulate genome packaging since it has been reported to interact with the NS5A protein, and NS5A is implicated in virion assembly, specifically in delivering the viral genomic RNA to the core protein for packaging [8,38,71]. To test this, we compared HCV RNA accumulation of wild-type full-length HCV RLuc reporter RNAs to those with defects in viral packaging (Figure 6).

Firstly, we measured the accumulation of wild-type J6/JFH-1 RLuc RNA (Figure 6A,B). Notably, while this RNA is assembly-competent, the addition of the large RLuc reporter gene is known to impair infectious particle production [48,72,73]. Nonetheless, in line with what we observed using the HCVcc system (Figure 1) and for the *core*-coding region containing subgenomic replicons (Figure 5), PCBP2 knockdown impaired viral RNA accumulation of the full-length wild-type RLuc reporter RNA (Figure 6A,B). Next, we introduced point mutations into the core-coding region (at residues 64–66, termed Core64–66), previously shown to abolish infectious particle production, but not core protein localization [6]. Additionally, we inserted eGFP into the NS5A domain III region, previously shown to reduce virion assembly (~50-fold) without impacting RNA replication [8,74,75]. While the former is thought to abolish viral assembly by disrupting a core-NS3 interaction necessary for virion assembly, the latter reduces viral assembly while keeping the core-coding region RNA sequence completely intact [6,75]. Interestingly, PCBP2 knockdown had no significant effect on the Core64–66 mutant (Figure 6C,D), while in the latter NS5A-GFP reporter RNA, only a small but significant decrease in luciferase activity was observed at the peak of replication (Figure 6E,F). Collectively, these results suggest that PCBP2 knockdown only modulates the accumulation of packaging-competent viral RNAs.

## 4. Discussion

**Figure 7:**
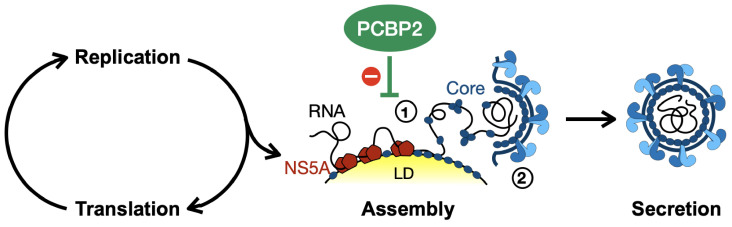
Model for PCBP2’s role in the HCV life cycle. HCV virion assembly is a two-step process whereby: (1) NS5A transfers the viral genome to the core protein at the surface of lipid droplets (LD); and (2) a protein complex involving NS2, NS3, NS4A and the structural proteins (core, E1 and E2) simultaneously assemble the nucleocapsid and envelope it as it buds into the ER lumen. Based on the data provided herein, our model suggests that PCBP2 normally inhibits the first stage of virion assembly, where the viral NS5A protein transfers the viral genome to the core protein.

Herein, we investigated the role of PCBP2 in the HCV life cycle. We found that PCBP2 knockdown did not affect HCV entry, IRES-mediated translation, genome stability or viral RNA replication. However, in line with the effects observed in the context of infectious virus (HCVcc), we observed an impairment in the accumulation of viral RNAs that could complete the early steps of the virion assembly process. Based on these findings, we propose a model where endogenous PCBP2 normally interferes with the transfer of the viral genomic RNA from NS5A to the core protein, thereby preventing premature virion assembly and may therefore indirectly impact viral translation and RNA replication (Figure 7).

Although HCV packaging and assembly are still incompletely understood, data to date suggest that it is a two-step process: (1) NS5A transfers the viral genome to the core protein at the surface of lipid droplets; and (2) a protein complex involving NS2, NS3, NS4A and the structural proteins (core, E1 and E2) simultaneously assembles the nucleocapsid and envelope as it buds into the ER lumen [4,5,6,8,9,71,76,77]. Based on our observation that PCBP2 knockdown only inhibits the accumulation of reporter RNAs with fully functional core and NS5A proteins, our data support a model where PCBP2 limits virion assembly during the first step, whereby NS5A transfers the viral genomic RNA to core. We favor this step rather than the overall process of virion assembly because ΔE1-p7 reporter RNAs were just as sensitive to PCBP2 knockdown as the WT full-length reporter RNA, whereas deletion or mutation of the core gene, or disruption of NS5A’s packaging efficiency, abolished this effect (Figure 5 and Figure 6). Since nucleocapsid formation occurs simultaneously with its envelopment, ΔE1-p7 viral RNAs cannot undergo appropriate packaging, but can still associate with the core protein, which is proposed to interact with numerous low-affinity sites across the viral RNA to mediate viral genome packaging [78,79,80].

While the precise molecular mechanism by which PCBP2 interferes with the core-NS5A interaction remains unclear, we envision that PCBP2 could mediate its effects on the viral life cycle either by interacting with the viral proteins themselves or through direct interactions with the viral RNA. Firstly, as previous studies reported that PCBP2 binds to NS5A in vitro and can co-immunoprecipitate exogenously overexpressed NS5A, it is possible that PCBP2 interferes with viral RNA transfer through direct interactions with the NS5A protein [38,81]. PCBP2 interactions with NS5A may directly block NS5A interactions with the viral RNA or core protein, or could interfere with the NS5A phosphorylation events necessary for the delivery of the viral genome to core [82]. Alternatively, it is possible that PCBP2’s interactions with the viral RNA may preclude NS5A or core from interacting with specific sites on the viral RNA. Notably, iCLIP analysis mapped the major PCBP2 binding site to the poly(U/UC) tract, a region of the genome known to be bound by NS5A; thus, it is possible that PCBP2 and NS5A may compete for binding to this region, or to other low affinity sites across the viral RNA important for genome packaging [40,78,80,83]. Interestingly, in addition to interactions with viral proteins, previous studies have shown that the HCV core protein can interact with the related cellular protein, hnRNPK, a known binding partner of PCBP2 [54,73,84]. Similarly to our findings herein, hnRNPK was found to suppress HCV particle production without affecting viral RNA replication [73]. Given that we mapped the PCBP2 sensitivity to the core protein, it is tempting to speculate that PCBP2 may exert its effects on genome packaging through interactions with hnRNPK and core. However, more research will be needed to further explore this possibility.

Importantly, our model does not require PCBP2 to play a direct role in translation or viral RNA replication to ultimately enhance intracellular viral protein expression or RNA accumulation. Our finding that PCBP2 has no direct effect on viral translation when assessed in isolation is consistent with prior studies that examined PCBP2’s effect on HCV IRES-mediated translation [31,32,33,35,36]. Furthermore, we found that PCBP2 was not necessary for viral RNA replication in subgenomic replicons that do not contain the *core* gene, which is consistent with a prior report that PCBP2 knockdown had no effect on the replication of a subgenomic HCV replicon that did not contain the *core* through *NS2* genes [37]. Moreover, PCBP2 knockdown had no effect on viral RNAs that were defective in viral genome assembly, suggesting that PCBP2 mediates its effects during viral genome packaging. While these results are largely in agreement with prior studies, our findings are in contrast with those reported by Masaki et al., who found that silencing PCBP2 reduced nascent viral protein synthesis in a stably infected Huh-7.5 cell line [34]. However, a direct effect on protein synthesis is not necessarily supported by their data, which suggests that PCBP2 knockdown both impairs viral translation and decreases viral RNA accumulation to a similar extent, thereby reducing the overall quantity of templates available for translation [34]. Interestingly, their data also suggest that PCBP2 knockdown did not reduce the rate of nascent viral RNA synthesis, suggesting that a similar number of viral replication complexes are present under PCBP2 knockdown and control conditions. This observation is compatible with the model proposed herein, as PCBP2 knockdown reduces the total intracellular RNA pool by promoting virion packaging, which is unlikely to disrupt established replication complexes in a persistently HCV-infected cell population. Moreover, since previous studies relied heavily on assays using small genome fragments (in vitro or in cells), purified proteins, and/or PCBP2 manipulation in infectious systems, these previous studies are consistent with our findings herein, which more specifically delineate the impact of PCBP2 on each step of the viral life cycle [38,39,41,42].

In conclusion, our results clarify the role of PCBP2 in the HCV life cycle and support a model where endogenous PCBP2 inhibits the early steps of virion assembly mediated by the core and NS5A proteins. By preventing viral genome sequestration by the core protein, PCBP2 indirectly promotes viral RNA retention in the translating/replicating pool, although its precise molecular mechanism of action still needs to be dissected. Nonetheless, PCBP2 exemplifies how a cellular RNA-binding protein can influence viral genomic RNA utilization and alter the balance of viral RNAs engaged in the different steps of the HCV life cycle.

## Data Availability

All data are contained within the article and Supplementary Materials.

## Supplementary Materials

The following supporting information can be downloaded at: https://www.mdpi.com/article/10.3390/v16081220/s1. Figure S1. Optimization of the anti-PCBP2 siRNA transfection. (A) Comparison between single-siRNA transfections and the 1:1 combination of both siRNAs (siPCBP2-mix). The error bars (below) represent the standard deviation from two independent experiments. (B) Analysis of PCBP2 knockdown over time, compared to the siCTRL condition. PCBP2/actin band densities were compared to the same day’s siCTRL condition (line graph, below); error bars represent the standard deviation from two independent replicates. Figure S2. PCBP2 knockdown does not affect Δcore-p7 RNA accumulation, but decreases ΔE1-p7 RNA accumulation. (A) Two days post-electroporation into Huh-7.5 cells, Δcore-p7 and (B) ΔE1-p7 RNA accumulation was assessed by Northern blot. (C) Δcore-p7 RNA accumulation and (D) ΔE1-p7 RNA accumulation was also assessed by RT-qPCR. Error bars represent the standard deviation of two independent Δcore-p7 replicates or three independent ΔE1-p7 replicates. *p*-values were determined by paired *t*-test (ns, not significant; ** *p* < 0.01).
