# Supplementary material for: Poly(rC)-Binding Protein 2 Does Not Directly Participate in HCV Translation or Replication, but Rather Modulates Genome Packaging
Source: Viruses. 2024 Jul 30;16(8):1220. doi: 10.3390/v16081220 (PMC11359930; doi:10.3390/v16081220)
Supplement: Supplementary file 1 [file viruses-3117738-supplementary.pdf › viruses-3117738-supplementary.pdf]

SUPPLEMENTARY FIGURES

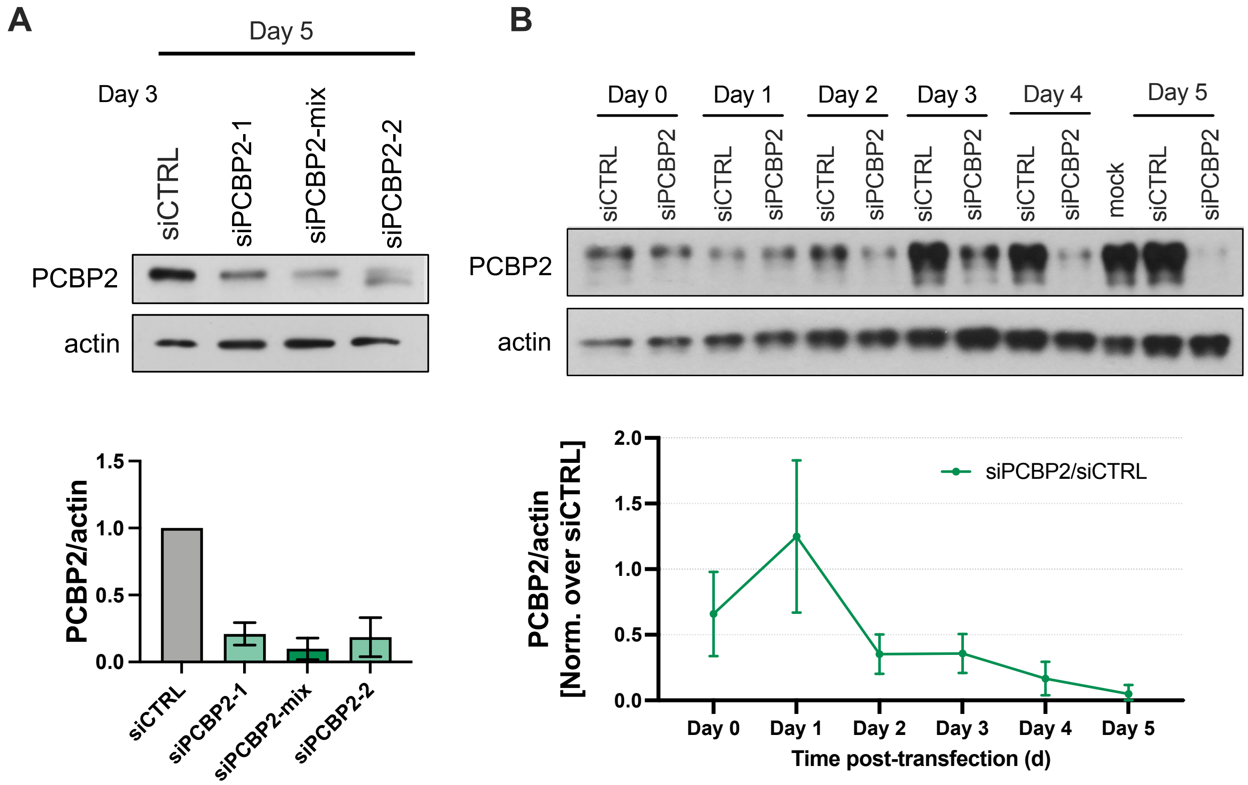

Figure S1. Optimization of the anti-PCBP2 siRNA transfection. (A) Comparison between single-siRNA transfections and the 1:1 combination of both siRNAs (siPCBP2-mix). The error bars (below) represent the standard deviation from two independent experiments. **(B)** Analysis of PCBP2 knockdown over time, compared to the siCTRL condition. PCBP2/actin band densities were compared to the same day's siCTRL condition (line graph, below); error bars represent the standard deviation from two independent replicates.

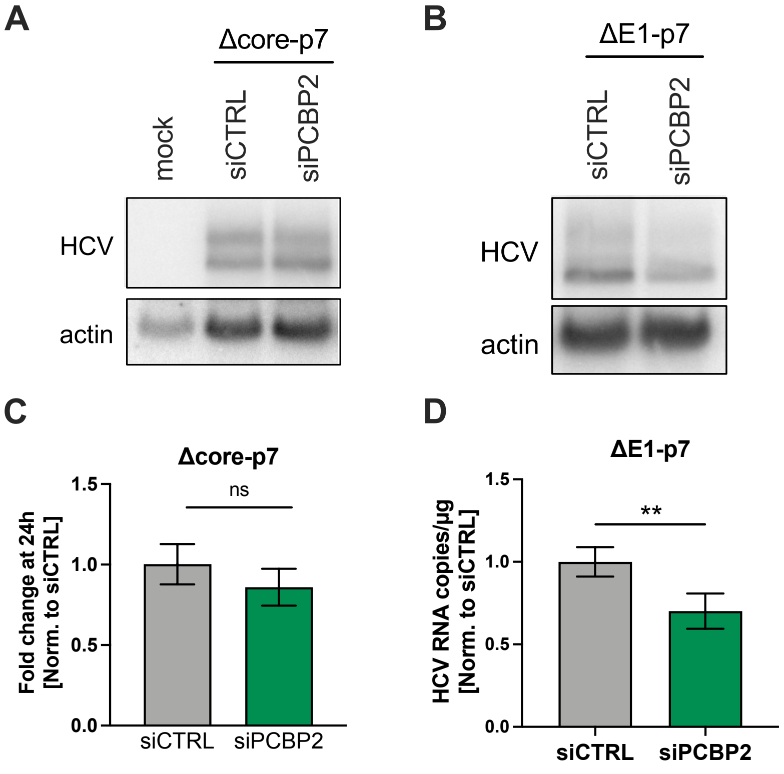

Figure S2. PCBP2 knockdown does not affect Δcore-p7 RNA accumulation, but decreases ΔE1-p7 RNA accumulation. (A) Two days post-electroporation into Huh-7.5 cells, Δcore-p7 and **(B)** ΔE1-p7 RNA accumulation was assessed by Northern blot. **(C)** Δcore-p7 RNA accumulation and **(D)** ΔE1-p7 RNA accumulation was also assessed by RT-qPCR. Error bars represent the standard deviation of two independent Δcore-p7 replicates or three independent ΔE1-p7 replicates. P-values were determined by paired t-test (ns, not significant; ** $p < 0.01$).
